# Adherence to International Guidelines in Pediatric Tonsillectomy: A National Survey among Brazilian Otolaryngologists *

**DOI:** 10.1055/a-2801-7896

**Published:** 2026-05-20

**Authors:** Manoel de Nobrega, Daniela Carvalho, José Faibes Lubianca Neto

**Affiliations:** 1 Pediatric Otorhinolaryngology Program, Department of Otorhinolaryngology and Head & Neck Surgery, Escola Paulista de Medicina, Universidade Federal de São Paulo (UNIFESP), São Paulo, SP, Brazil; 2 Department of Otolaryngology, Head and Neck Surgery, University of California, San Diego; 3 Hearing Loss and Cochlear Implant Program, Rady Children's Hospital, San Diego, CA, United States; 4 Department of Sugical Clinic, Federal University of Health Sciences, Division of Pediatric ENT at Santo Antonio Children's Hospital, Porto Alegre, RS, Brazil; 5 Universidade Federal do Rio Grande do Sul (UFRGS), Porto Alegre, RS, Brazil

**Keywords:** tonsillectomy, children, otolaryngology, complications, clinical guidelines

## Abstract

**Introduction:**

The present study aimed to evaluate the adherence of Brazilian otolaryngologists to key recommendations from contemporary international tonsillectomy guidelines, particularly the 2019 Clinical Practice Guideline by Mitchell et al.

**Objective:**

To evaluate how Brazilian otorhinolaryngologists perform tonsillectomies and manage potential postoperative complications.

**Methods:**

An online questionnaire was developed and completed by otorhinolaryngologists affiliated with relevant medical associations.

**Results:**

Revealed that 40.4% of surgeons had over 20 years of experience, indicating substantial expertise. Primary indications for tonsillectomy included snoring, hypertrophy, and recurrent tonsillitis. Diagnostic criteria for acute bacterial tonsillitis and obstructive sleep apnea were analyzed, with 35% of respondents considering episode frequency a key criterion. Polysomnography was requested in specific cases, reflecting concerns about respiratory severity.

**Conclusion:**

The present study provides the first comprehensive analysis of the adoption of international guidelines for tonsillectomy in Brazil. We conclude that, although the Brazilian otolaryngology community has adopted key recommendations regarding diagnosis and postoperative care, deeply entrenched deviations persist in technical aspects and intraoperative medication. These latter aspects represent opportunities for educational interventions, resource mobilization, and the development of adapted national guidelines that take local realities into account, with the ultimate goal of standardizing and improving the quality of care for all children undergoing tonsillectomy in the country.

## Introduction

Annually, in the United States, over 500 thousand children under 15 years of age undergo tonsillectomy or adenotonsillectomy, 1 accounting for more than 15% of all surgical procedures in this age group. 1 2 The primary indications for tonsillectomy are obstructive sleep apnea and recurrent throat infections. Although generally considered safe and straightforward, tonsillectomy carries risks that vary in frequency and severity. 1

Tonsillectomy complications are more common than with other routine pediatric surgeries: 2.7% of children are readmitted within 30 days, and 12.4% present to emergency departments, often due to hemorrhage. Fatalities occasionally occur. 3

Therefore, the present study aims to map the current tonsillectomy practices among Brazilian otolaryngologists, specifically evaluating the degree of alignment with the 2019 international guideline recommendations by Mitchell et al. (2019) 4 By doing so, it seeks to identify areas of excellent adherence and, crucially, to highlight specific practice patterns in which implementation gaps exist, thus informing future educational and protocol-development initiatives in the Brazilian healthcare context.

## Methods

The present study was approved by the Research Ethics Committee of Universidade Federal de São Paulo (UNIFESP) under protocol number 4.410.567.

Following the methodology of Bezdjian et al. (2018) 5 , we developed an online questionnaire using Google Forms (Google, LLC, https://docs.google.com/forms/d/e/1FAIpQLSccq-Ou060hZl-E5E4BbI4gsuiXo5X7d36faHT63VA6h1eg3A/viewform ). The survey addressed surgical techniques (including aseptic protocols, operative methods etc.) and complications and was completed by otolaryngologists affiliated with Academia Brasileira de Otorrinolaringologia Pediátrica and/or the Otolaryngology Scientific Department of Sociedade Brasileira de Pediatria.

The questionnaire was validated by the authors and anchored in established guidelines 4 to minimize ambiguities. Responses were collected anonymously and voluntarily. This target group was selected as it represents the majority of Brazilian otolaryngologists with a strong focus on pediatric care. Notably, Brazil lacks formal subspecialty certification in pediatric otolaryngology. The survey was designed to reflect actual surgical practice and was jointly developed and reviewed by all authors.

It is important to emphasize that the current study, by its nature as a self-report questionnaire, is subject to inherent biases, such as recall bias and social desirability bias. Specifically, data on complications, such as bleeding rates, represent estimates provided by surgeons based on their clinical experience, and not objective measures obtained through chart audits. Therefore, it is not possible to calculate precise incidence rates. The definition of terms such as *massive bleeding* was left to the respondent's interpretation, which may explain the variation in reported rates when compared with the literature that uses standardized definitions.

## Statistical Analysis

All variables were initially analyzed descriptively. For qualitative variables, absolute and relative frequencies were calculated. The Z-test for two proportions was used to compare whether response proportions between two variables and/or their levels were statistically significant. 6 7 8 9 10 Statistical analyses were performed using the IBM SPSS Statistics for Windows (IBM Corp.), Minitab 21.2 (Minitab, LLC), and Excel Office 2010 (Microsoft Corp.). The Chi-squared test for independence was employed to assess whether two variables and their levels exhibited statistical dependence (association). 10

## Results

The questionnaire was sent to 252 Brazilian otolaryngologists who treat children, with 184 responses received (response rate: 73%). Results are presented under the following topics: Indications; prospective care; surgical technique; and postoperative care.

### Profile of Brazilian Otolaryngologists

Surgeons with more than 20 years of experience were the most prevalent group (40.4%), a statistically significant proportion compared with other cohorts ( Table 1 ).

**Table 1:** How many years have you been operating?

P1	N	%	*p* -value
**> 20 years**	**74**	**40.4%**	**Reference**

#### Indications ( Table 2 )

**Table 2:** Distribution of tonsillectomy practices and complications according to the surgeon's experience level

		≤ 10 years		11–20 years		> 20 years		*p* -value
		N	%	N	%	N	%	
What are the most common indications for tonsillectomy in your practice?								
Peritonsillar abscess	Never	5	9.4%	8	14.3%	8	10.8%	0.301
	< 25%	40	75.5%	43	76.8%	50	67.6%	
	25–50%	5	9.4%	4	7.1%	13	17.6%	
	50–75%	0	0.0%	1	1.8%	0	0.0%	
	> 75%	3	5.7%	0	0.0%	3	4.1%	
Antibiotic allergies/intolerance	Never	40	75.5%	39	69.6%	57	77.0%	0.771
	< 25%	10	18.9%	15	26.8%	16	21.6%	
	25–50%	2	3.8%	1	1.8%	1	1.4%	
	50–75%	1	1.9%	1	1.8%	0	0.0%	
Recurrent tonsillitis	Never	0	0.0%	0	0.0%	1	1.4%	0.404
	< 25%	18	34.0%	27	48.2%	38	51.4%	
	25–50%	22	41.5%	22	39.3%	25	33.8%	
	50–75%	8	15.1%	5	8.9%	8	10.8%	
	> 75%	5	9.4%	2	3.6%	2	2.7%	
Caseum/halitosis	Never	5	9.4%	6	10.7%	12	16.2%	0.805
	< 25%	38	71.7%	44	78.6%	51	68.9%	
	25–50%	8	15.1%	5	8.9%	9	12.2%	
	50–75%	2	3.8%	1	1.8%	2	2.7%	
Hypertrophy	Never	6	11.3%	2	3.6%	2	2.7%	0.009
	< 25%	10	18.9%	10	17.9%	8	10.8%	
	25–50%	24	45.3%	24	42.9%	28	37.8%	
	50–75%	2	3.8%	15	26.8%	21	28.4%	
	> 75%	11	20.8%	5	8.9%	15	20.3%	
PFAPA (periodic fever, aphthous stomatitis, pharyngitis, and adenitis)	Never	15	28.3%	19	33.9%	24	32.4%	0.482
	< 25%	35	66.0%	35	62.5%	45	60.8%	
	25–50%	1	1.9%	1	1.8%	3	4.1%	
	50–75%	0	0.0%	1	1.8%	2	2.7%	
	> 75%	2	3.8%	0	0.0%	0	0.0%	
Snoring/ Obstructive Sleep Apnea Syndrome	Never	0	0.0%	0	0.0%	2	2.7%	0.092
	< 25%	5	9.4%	4	7.1%	15	20.3%	
	25–50%	26	49.1%	27	48.2%	26	35.1%	
	50–75%	6	11.3%	13	23.2%	16	21.6%	
	> 75%	16	30.2%	12	21.4%	15	20.3%	
What intraoperative complications have you had?								
Anaphylactic shock	< 25%	0	0.0%	3	5.4%	5	6.8%	0.169
	No case	53	100.0%	53	94.6%	69	93.2%	
Death	< 25%	2	3.8%	0	0.0%	3	4.1%	0.320
	No case	51	96.2%	56	100.0%	71	95.9%	
Other complications	No case	37	69.8%	32	57.1%	48	64.9%	0.178
	< 25%	16	30.2%	22	39.3%	24	32.4%	
	25–50%	0	0.0%	0	0.0%	2	2.7%	
	> 75%	0	0.0%	2	3.6%	0	0.0%	
Cardiopulmonary arrest	< 25%	1	1.9%	1	1.8%	12	16.2%	0.002
	No case	52	98.1%	55	98.2%	62	83.8%	
Massive bleeding	No case	18	34.0%	17	30.4%	19	25.7%	0.681
	< 25%	34	64.2%	38	67.9%	53	71.6%	
	25–50%	1	1.9%	0	0.0%	1	1.4%	
	50–75%	0	0.0%	0	0.0%	1	1.4%	
	> 75%	0	0.0%	1	1.8%	0	0.0%	
Do you prescribe antibiotics postoperatively?	In selected cases	21	39.6%	26	46.4%	33	44.6%	0.849
	Never	11	20.8%	13	23.2%	18	24.3%	
	Always	21	39.6%	17	30.4%	23	31.1%	
Do you prescribe analgesics?	sometimes	1	1.9%	1	1.8%	2	2.7%	0.925
	Always	52	98.1%	55	98.2%	72	97.3%	
Have you ever had late bleeding (> 24 hours after surgery)?	< 25%	52	98.1%	55	98.2%	69	93.2%	0.553
	25–50%	1	1.9%	0	0.0%	1	1.4%	
	50–75%	0	0.0%	0	0.0%	1	1.4%	
	> 75%	0	0.0%	1	1.8%	3	4.1%	

The most frequent indications for tonsillectomy were snoring (most common) followed by hypertrophy and recurrent tonsillitis.

The main criteria used for the diagnosis of acute bacterial tonsillitis were the presence of tonsillar exudate (most cited), fever above 38 °C, and cervical adenopathy.

For 35% of respondents, recurrent pharyngotonsillitis was defined as: 7 episodes in the past year, or 5 episodes/year for 2 years, or 3 episodes/year for 3 years. While 35% of surgeons adhered to the 2019 guideline criteria 4 for recurrent pharyngotonsillitis, discrepancies in other aspects (e.g., intraoperative dexamethasone use) suggested implementation barriers (e.g., lack of institutional protocols or drug access).

The most common factors considered when suspecting obstructive sleep apnea are poor academic performance, growth retardation, and behavioral problems.

Polysomnography is requested mainly for children with comorbidities (45.4%) and when the physical examination does not suggest respiratory severity (23.5%). It is never requested in 23% of cases.

Obstructive sleep apnea (OSA) suspicion factors:

#### Preoperative Care ( Table 2 )

In 43.7% of cases, sedatives/hypnotics are never used preoperatively, and in 20.8% of cases, they are always used. The most commonly used sedative/hypnotic in the preoperative period is midazolam (77.3% of cases). Prophylactic antibiotic therapy is never used in 73.8% of cases.

#### Surgical Technique ( Table 2 )

The absolute majority (94.5%) of surgeons operate under direct vision. The most commonly used surgical technique is cold dissection (76%). The predominance of cold dissection in the current study contrasts with global data, according to which techniques such as coblation and diathermy are more frequent (Spencer et al., 2025 11 ). This difference may be related to the availability of resources or institutional preferences.

Surgeons request intensive care unit (ICU) care for patients with comorbidities in 46.4% of cases, and it is never requested by 33.9% of surgeons.

Massive bleeding is the main occurrence of intraoperative complications, occurring in less than 25% of cases for 125 surgeons (95.6%). It is crucial to note that these are perceived and self-reported rates by the surgeons. The definition of *massive bleeding* was not standardized in the questionnaire, being interpreted subjectively by each participant, which may influence the reported values.

#### Postoperative Care ( Table 2 )

For 80.9% of surgeons, patients without comorbidities are discharged on the same day. For patients with comorbidities (e.g., syndromic, morbid obesity, severe OSAS), 81.4% of surgeons usually discharge patients on the following day.

The diet recommended by 40.4% of surgeons is initially a cold liquid diet for 3 to 4 days, and then a cold liquid paste for another 3 to 4 days. Dietary guidance is recommended for 7 days for 76.1% of surgeons.

Antibiotics are prescribed postoperatively by 33.3% of surgeons. The most commonly used antibiotic postoperatively is amoxicillin (54.7%) and amoxicillin with potassium clavulanate (28.5%).

The vast majority of surgeons (97.8%) always prescribe analgesics. The option of using 2 analgesics alternately is adopted by 58.5% of surgeons. Dipyrone is the first choice for 93.8% of surgeons, and paracetamol is the second choice for 81%.

Narcotics in the postoperative period are not used by 119 surgeons (65%), and midazolam is the main narcotic used by 58 surgeons (77.3%).

Antiinflammatories in the postoperative period are always used by 23% of surgeons. The use of hormonal antiinflammatories is used by 38.2% of surgeons.

The return of the patient 7 days after surgery is the rule for 79.2% of surgeons.

Primary bleeding occurred in less than 25% of the cases operated on by all surgeons interviewed. Delayed bleeding occurred in less than 25% of the cases operated on by 96.2% of surgeons interviewed. The main late complication (other than bleeding) was readmission for intravenous hydration for 56.8% of surgeons. Again, it is crucial to note that these are perceived and self-reported rates by the surgeons. The definition of *massive bleeding* was not standardized in the questionnaire, being interpreted subjectively by each participant, which may influence the reported values.

The time since graduation had a statistically significant relationship with only two questions: “what are the most common indications for tonsillectomy in your practice?” and “hypertrophy” ( *p* -value = 0.009) and “what intraoperative complications have you had?” and “cardiorespiratory arrest” ( *p* -value = 0.002).

In relation to hypertrophy, analyzing the main responses, the rate of occurrence between 25 to 50% was 45.3% for people with ≤ 10 years since graduation, 42.9% for 11 to 20 years, and 37.8% for > 20 years. The rate of occurrence between 50 to 75% was 3.8%, 26.8% and 28.4%, respectively, in the groups and time they have been operating. Finally, the rate of more than 75% was 20.8% for those ≤ 10 years since graduation, 8.9% for those 11 to 20 years and 20.3% for those > 20 years.

In relation to cardiorespiratory arrest, the rate of less than 25% occurrence was 1.9% for those ≤ 10 years since graduation, 1.8% for those 11 to 20 years, and 16.2% for those >20 years. As for the rate of no reported cases, the rates were 98.1%, 98.2%, and 83.8%, respectively.

## Discussion

The present survey provides a unique analysis of the integration of evidence-based guidelines into Brazilian ear, nose, and throat (ENT) practice, revealing a heterogeneous landscape of adherence. The results paint a detailed picture where commendable alignment in several areas contrasts with notable disparities in others, a dichotomy that likely reflects profound local contextual challenges.

### Profile of Brazilian Otorhinolaryngologists

The demographic profile of Brazilian otorhinolaryngologists in our study revealed that surgeons with over 20 years of practice constituted the most prevalent group. This finding contrasts with the study by Clark et al. (2018), 12 in which respondents with less than 10 years of training represented the majority (41.5%). Their questionnaire, administered through REDCap (Vanderbilt University), examined perioperative management approaches for post-tonsillectomy bleeding cases, comparing factors such as pediatric otolaryngology training, practice regions, and years of experience.

#### Indications

Regarding surgical indications, our results demonstrate concordance with Greig's 2017 study, in which obstructive sleep apnea (frequently combined with adenoidectomy) and recurrent pharyngotonsillitis emerged as primary indications for tonsillectomy. 13

Only 35% of surgeons adopted the 2019 guidelines for recurrent tonsillitis, 4 indicating a significant gap in implementation.

In our cohort, the most frequently considered factors for suspecting obstructive sleep apnea included poor academic performance, growth delay, and behavioral issues. The pattern of polysomnography requests—predominantly for children with comorbidities (45.4%) or when physical examination failed to suggest respiratory severity (23.5%)—aligns with the 2019 guidelines 4 for testing children under two years or those presenting with specific clinical conditions such as obesity, Down syndrome, craniofacial anomalies, or neuromuscular disorders.

#### Preoperative

The preoperative management analysis revealed a notable deviation from the 2019 guidelines 4 for intraoperative intravenous dexamethasone administration, a practice not adopted by Brazilian otorhinolaryngologists in our study population. This discrepancy warrants further investigation into potential barriers to guideline implementation in the Brazilian healthcare context.

#### Surgical Technique

The predominance of cold dissection (76%) represents a significant deviation from global trends and techniques frequently endorsed by evidence of superiority in some outcomes, such as bipolar diathermy for primary hemorrhage. This discrepancy may be attributed to barriers to access to technology or to established surgical training paradigms.

Major intraoperative bleeding represented the most common complication. These findings contrast with data from Mowatt et al. (2006), 14 according to which bipolar diathermy demonstrated superior outcomes for primary hemorrhage prevention(odds ratio [OR]: 0.13; 95% credible interval [CrI]: 0.03–0.51), while coblation techniques showed increased risk of secondary hemorrhage requiring surgical reintervention (OR: 33.82, 95%CrI: 1.25–5,676.00). The persistence of cold dissection as the primary technique in our study may reflect regional differences in equipment availability or surgical training paradigms.

#### Postoperative Care

Postoperative care patterns in our study demonstrated several noteworthy findings. The discharge timing—same-day for non-comorbid patients (80.9%) and next-day for complex cases (81.4%)—corresponds with that reported in the established literature, 13 recommending overnight observation for children under three years or those with severe OSA. The 2019 guideline 4 specifically advises overnight monitoring for patients exhibiting an apnea-hypopnea index ≥ 10 events/hour or oxygen saturation below 80%.

Dietary protocols predominantly featured initial cold liquids (3–4 days) followed by cold pureed foods (3–4 days), totaling 7 days of modified diet regardless of surgical indication. This contrasts with literature documenting faster return to normal eating patterns in children undergoing tonsillectomy for obstructive versus infectious indications. 15

The fact that 73.8% of respondents did not use prophylactic antibiotics is perfectly aligned with the strong recommendation of the 2019 guidelines, 4 demonstrating the successful incorporation of evidence against a historically common practice.

Most surgeons in the current study always prescribe analgesics, with more than half of the interviewees choosing to use 2 analgesics alternately, with dipyrone as the first choice and paracetamol as the second choice. This conduct is in accordance with that proposed by the 2019 guidelines, 4 which recommends ibuprofen, paracetamol, or both for pain control after tonsillectomy. They also advise that the physician should inform patients and caregivers on the importance of post-tonsillectomy pain management as part of the perioperative education process and reinforce this advice at the time of surgery with reminders about the need to anticipate, reassess, and adequately treat pain after surgery.

Postoperative narcotic is not used by most respondents. When prescribed, midazolam is the primary narcotic used. In the literature, codeine is contraindicated due to reports of postoperative death from respiratory suppression. 13 Postoperative recovery is often associated with high levels of pain and severe functional limitations. There is currently no consensus on pain management regimens. Furthermore, a recent Food and Drug Administration (FDA) warning regarding narcotic use has created further uncertainty regarding appropriate pain management regimens. 16 In the same study cited above, post-tonsillectomy patients and their parents found a prescription for acetaminophen-hydrocodone to be therapeutically valuable, and many found it necessary in the management of postoperative pain. 16 The 2019guidelines 4 recommend that physicians should not administer or prescribe codeine, or any medications containing codeine, after tonsillectomy in children younger than 12 years.

Only a small proportion of respondents reported using non-steroidal or steroidal antiinflammatory medications. In the context of this study, the term *hormonal antiinflammatory drugs* was used to refer to corticosteroids (e.g., dexamethasone, prednisone), distinguishing them from non-steroidal antiinflammatory drugs (NSAIDs). The low reported utilization rate (38.2%) is noteworthy, especially considering the strong recommendation in the 2019 guidelines 4 for the use of intraoperative dexamethasone to reduce nausea, vomiting, and pain. This finding warrants careful consideration in light of conflicting evidence from recent studies. Hsueh et al. (2018) 17 demonstrated an association between NSAID/steroid use and increased risk of readmission or reoperation, suggesting caution in their postoperative application. However, Miyamoto et al. (2018) 18 presented contrasting data, finding no statistically significant difference in reoperation rates between patients who received steroids on the day of tonsillectomy (1.8%, *n* = 15) versus those who did not (1.5%, *n* = 79), with an adjusted odds ratio of 0.81 (95%CI: 0.45–1.43; *p* = 0.46). Their analysis similarly revealed no significant associations in either adult (OR: 0.73; 95%CI: 0.38–1.38; *p* = 0.33) or pediatric subgroups (OR: 1.18; 95%CI: 0.34–4.11; *p* = 0.80). Based on these comprehensive findings, the authors concluded that steroid administration on the day of tonsillectomy showed no association with increased risk of reoperation due to bleeding. This discrepancy between studies highlights the ongoing need for careful patient selection and individualized decision-making when considering anti-inflammatory use in the postoperative period. The conservative approach adopted by Brazilian otolaryngologists in our study may reflect both this clinical uncertainty and potential concerns about medication availability or cost in the local healthcare setting.

The low incidence of primary bleeding observed in our study aligns with the findings of Mahadevan et al. (2016) 19 , who reported a primary postoperative bleeding rate of 0.5% (95%CI: 0.3–0.7%), with a combined rate of primary and secondary post-tonsillectomy bleeding of 4.3% (95%CI: 3.8–5.0%). Their study also found that 6.3% (95%CI: 5.6–7.0%) of patients returned with postoperative complications within one month of surgery.

The higher rates of bleeding reported here—both intraoperative ( *massive* ) and postoperative—compared with objective data in the literature likely reflect methodological limitations rather than a true higher incidence. The absence of a standardized definition for *massive bleeding* , combined with recall bias for dramatic events, means these figures represent surgeons' risk perception rather than precise epidemiological measures.

Our analysis of cold dissection technique and delayed bleeding revealed remarkably low incidence rates. This finding gains particular significance when compared with other surgical approaches. Blanchford and Lowe (2013) 20 demonstrated significantly increased bleeding rates for diathermy dissection (adjusted OR: 2.47, *p* < 0.0001) and coblation tonsillectomy (adjusted OR: 3.07, *p* < 0.0001) when compared with the reference category of cold dissection tonsillectomy. Further supporting our observations, Brkić et al. (2017) 21 conducted a comparative study between “hot” (monopolar/bipolar forceps dissection with hemostasis) and combined technique (cold steel dissection with monopolar/bipolar hemostasis) in relation to delayed hemorrhage occurrence. Their results showed postoperative bleeding in 46 patients (4.23%), with a striking distribution: 45 patients (4.88%) experienced postoperative hemorrhage following the combined technique, while bleeding occurred in only 1 patient (0.6%) after the hot technique. This represents an approximately 8-fold lower bleeding rate with the hot technique ( *p* = 0.012), providing additional context for understanding the bleeding risks associated with different surgical approaches.

The most frequent late complication (excluding bleeding) was hospital readmission for intravenous hydration. This finding is supported by Johnson et al. (2018) 22 , who analyzed 9,079 children undergoing tonsillectomy (with or without adenoidectomy), of whom 327 (3.6%) required readmission. The readmitted children had a mean age of 5.0 years, with 51% being female. The most common readmission diagnoses were dehydration (47%), hemorrhage (26%), and pain (16%). The mean time to readmission was 7.3 days, with specific averages of 6.3 days for bleeding, 4.5 days for pain, and 4.1 days for dehydration-related readmissions.

A relevant question is whether the participants were aware of the specific guideline. Although the question was not directly asked, the high adherence to some recommendations (such as not using antibiotic prophylaxis) suggests widespread knowledge of its premises, possibly through continuing medical education. On the other hand, the marked divergences in other aspects (such as the criteria for recurrent tonsillitis) indicate that the mere existence of the guideline does not guarantee its uniform implementation, and there may be barriers such as the lack of institutional protocols or the influence of traditional practices.

## Conclusion

The present study provides the first comprehensive analysis of the adoption of international tonsillectomy guidelines in Brazil. We conclude that, while the otolaryngology community in Brazil demonstrates assimilation of crucial recommendations in diagnosis and postoperative care, deeply ingrained deviations persist in technical aspects and intraoperative medication. These latter aspects represent target opportunities for educational interventions, advocacy for resources, and the development of adapted national guidelines that consider local realities, with the ultimate goal of standardizing and improving the quality of care for all children undergoing tonsillectomy in the country.
